# Repeated crack healing in MAX-phase ceramics revealed by 4D *in situ* synchrotron X-ray tomographic microscopy

**DOI:** 10.1038/srep23040

**Published:** 2016-03-14

**Authors:** Willem G. Sloof, Ruizhi Pei, Samuel A. McDonald, Julie L. Fife, Lu Shen, Linda Boatemaa, Ann-Sophie Farle, Kun Yan, Xun Zhang, Sybrand van der Zwaag, Peter D. Lee, Philip J. Withers

**Affiliations:** 1 Department of Materials Science and Engineering, Delft University of Technology, Mekelweg 2, 2628 CD, Delft, The Netherlands; 2 School of Materials, Manchester University M13 9PL, UK; 3 Swiss Light Source, Paul Scherrer Institut, 5232 Villigen PSI, Switzerland; 4 Faculty of Aerospace Engineering, Delft University of Technology, Kluyverweg 1, 2629 HS, Delft, The Netherlands; 5 Research Complex at Harwell, Didcot, Oxfordshire, OX11 0FA, UK

## Abstract

MAX phase materials are emerging as attractive engineering materials in applications where the material is exposed to severe thermal and mechanical conditions in an oxidative environment. The Ti_2_AlC MAX phase possesses attractive thermomechanical properties even beyond a temperature of 1000 K. An attractive feature of this material is its capacity for the autonomous healing of cracks when operating at high temperatures. Coupling a specialized thermomechanical setup to a synchrotron X-ray tomographic microscopy endstation at the TOMCAT beamline, we captured the temporal evolution of local crack opening and healing during multiple cracking and autonomous repair cycles at a temperature of 1500 K. For the first time, the rate and position dependence of crack repair in pristine Ti_2_AlC material and in previously healed cracks has been quantified. Our results demonstrate that healed cracks can have sufficient mechanical integrity to make subsequent cracks form elsewhere upon reloading after healing.

In recent years, new types of engineering materials have been developed that can repair internal crack and creep damage autonomously using healing mechanisms based on the physico-chemical nature of the material^1^. The application of these so called ‘self-healing’ materials has the potential to drastically increase the durability and reliability of structural components. For applications that require structural integrity at high temperatures, intrinsic (i.e. without the need to introduce discrete ‘foreign’ healing entities) self-healing ceramics would be ideally suited. Recently, a new class of ternary ceramics, known as MAX-phase metallo-ceramics, was found to have the unique ability to fully, and sometimes even repeatedly, heal cracks in a completely autonomous manner when exposed for sufficiently long times to intended high use temperatures in an oxidative gaseous environment^2^,^3^,^4^,^5^,^6^. These ceramics are composed of layered compounds with a M_n+1_AX_n_ configuration^7^ where M is an early transition metal, A is most commonly a group IIIA or IVA element (typically Al or Si) and X is either C or N. Due to its layered structure, a MAX phase material exhibits a unique combination of mechanical, thermal and electric properties^7^,^8^,^9^,^10^,^11^,^12^. The high thermal conductivity also makes these ceramics thermal shock resistant. Their static strength is maintained up to high temperatures, above which creep becomes the limiting factor^7^,^11^,^12^. Further, dislocations can multiply and glide on the basal planes of the hexagonal lattice^13^, while plastic deformation of polycrystalline MAX phase material typically occurs by a combination of kink and shear band formation, together with the delamination of lamellar grains^7^. In contrast to many other ceramics, MAX phase materials are tough and therefore damage tolerant and also easily machinable^14^.

In this work monolithic Ti_2_AlC MAX phase material was studied. The crystalline unit cell of Ti_2_AlC contains two sub units^11^. The octahedral Ti_2_C layers are interrupted by layers of pure Al, which forms a Ti_2_C-Al-Ti_2_C-Al layered structure. The space group of Ti_2_AlC is P6_3_/mmc with lattice parameters of a = 3.04 Å and c = 13.60 Å.

The self-healing behavior is due to oxidation reactions creating products that bond well to the crack faces and fill cracks with strong reaction product^5^. Cracks in Ti_2_AlC can be fully repaired due to the formation of fine-grained Al_2_O_3_ and some TiO_2_ within the crack gap upon high temperature oxidation^4^,^15^. While Al and some Ti are consumed during oxidation, the resulting non-stoichiometric Ti_2_AlC phase retains its hexagonal crystal structure^16^. In the Ti_2_AlC ceramic the outward diffusion of the weakly bonded Al atoms is much faster than that of the more covalently bonded Ti atoms in the Ti_2_AlC structure^5^,^7^,^17^,^18^,^19^. This difference in kinetics leads to the formation of small α-Al_2_O_3_ grains at the ledges of the fractured lamellar Ti_2_AlC grains as well as on the hexagonal basal surfaces ensuring a good adhesion with the parent matrix^17^. This good adhesion in combination with a minimal mismatch in thermal expansion coefficient and stiffness with the matrix is believed to be the cause of the complete restoration of the tensile strength provided the crack is adequately filled^4^,^5^,^17^. The stiffness of the healing product, mainly Al_2_O_3_^20^, is somewhat higher than that of Ti_2_AlC^21^, i.e. 400 versus 280 GPa. Hence, upon mechanical re-loading, a stress concentration would be expected to occur in the healed zone of these MAX phases and cracking would be expected to follow the path of previously healed cracks, unless the local healing is of a very high quality. The thermal expansion of α-Al_2_O_3_ (7.5–9.6 × 10^−6^ K^−1^)^22^, is about the same as that of Ti_2_AlC (8.2 × 10^−6^ K^−1^)^8^. This implies that little to no residual stress is generated in the oxide when the healed material is cooled down from the oxidation temperature.

*In situ* 4D (three spatial dimensions plus time) observation of crack formation and subsequent filling of the crack by a load bearing reaction product is crucial for understanding the self-healing behavior. Furthermore, quantification of the spatial and temporal dependence is needed to validate and develop new micromechanical models for crack healing currently under development. While the challenge of *in situ* observation and (low level) quantification of closing and healing of cracks were already extremely demanding for (polymeric) materials that fail and heal at room temperature^23^, the experimental challenges become orders of magnitude more complex for (ceramic) materials that operate and heal at high temperatures. Until now, it has not been possible to directly observe the crack filling in high temperature ceramics. Thus it has also not been possible to monitor the crack repair or to establish the integrity of the repair. This is true not only for cracks formed in the pristine material, but also for cracks passing through a previously healed region. 4D X-ray tomographic microscopy using the high flux and brilliance of synchrotron X-rays is now a powerful tool for imaging the spatial and temporal evolution of microstructures from macroscopic to submicroscopic scales within a variety of materials^24^,^25^,^26^,^27^,^28^,^29^,^30^,^31^.

## Results

### 4D X-ray tomographic microscopy and thermomechanical testing

**Figure 1:**
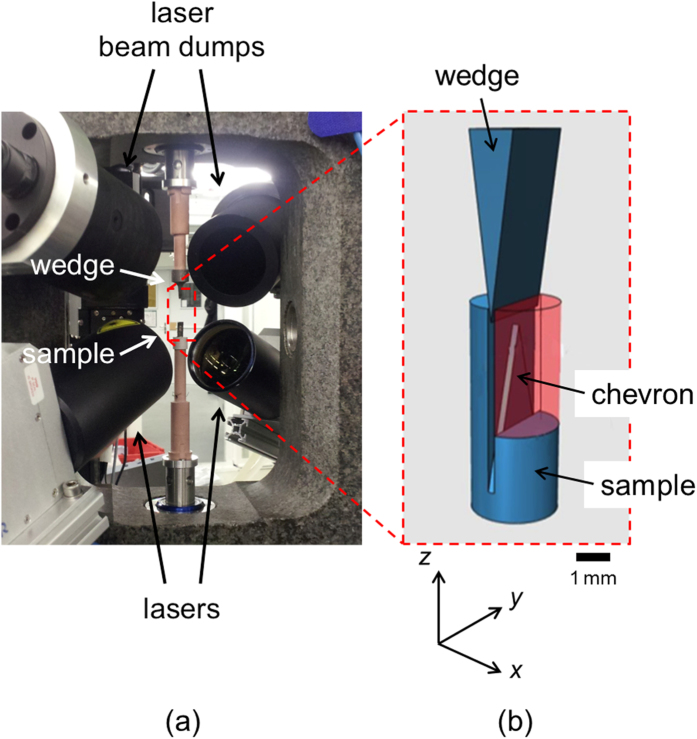
Thermomechanical testing rig for *in situ* 4D X-ray tomographic microscopy. (**a**) Image of the mechanical testing rig incorporating the laser-based heating system mounted at the TOMCAT beamline; the sample stage and wedge setup are also visible. (**b**) Sample and wedge configuration; the arrow indicates the chevron where the cracks are generated and healed.

The current study utilizes the non-destructive 3D imaging capability of tomography to obtain the first 4D observations of multiple cracking and self-healing cycles at high temperature. To observe the complex phenomena occurring both during crack growth and in subsequent crack healing both *in situ* and with time requires a means of applying mechanical load and high temperature simultaneously whilst rotating the sample and capturing 4D information with a spatial resolution high enough and a temporal resolution fast enough to resolve both the physical change in the microstructure and the dynamics of the processes. To accomplish the first part, an ultra-high precision mechanical testing rig^32^ was combined with a laser-based heating system^33^ as shown in Fig. 1. Cylindrical Ti_2_AlC samples having a diameter of 2.6 mm and 7 mm length were machined with chevron-notch geometry and the controlled displacement of a wedge was used to drive the crack (see Fig. 1b). Given the geometry of the sample and the wedged loading configuration the cracks are expected to form at either of the two intercepts between the triangular central section and the sides of the slot terminating the chevron.

### Crack initiation and healing

**Figure 2:**
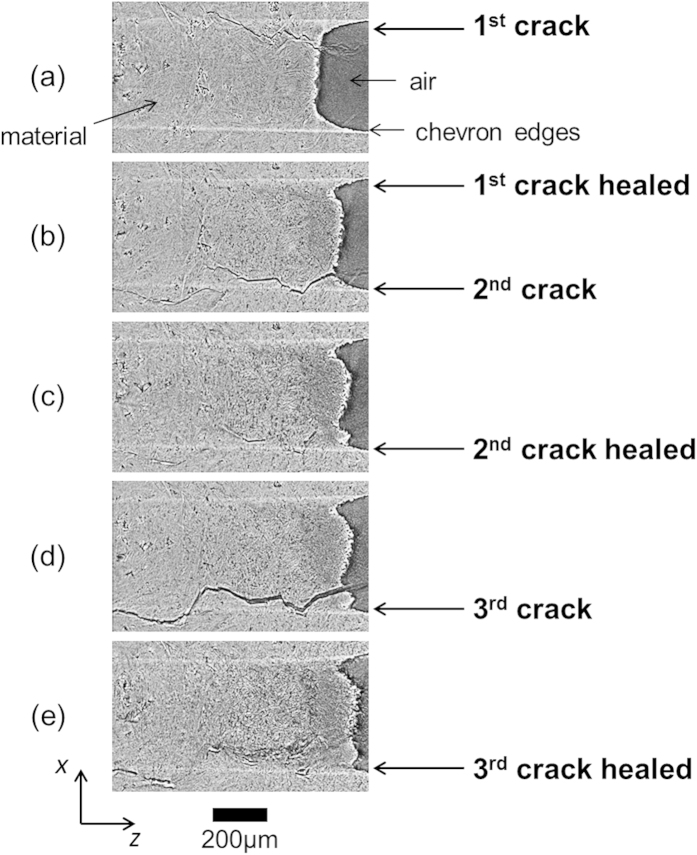
Virtual 2D sections through the tomographic datasets showing the sequence of crack growth and healing steps in Ti_2_AlC at 1500 K in air. Mid-section of the sample: (**a**) first crack, (**b**) second crack and healed first crack, (**c**) healed second crack, (**d**) third crack and reopening of healed second crack, (**d**) healed third crack.

Initial crack growth was performed at a temperature of 1000 K (see Supplementary information Video S1: A sequence of successive planar tomographic sections of the *initial crack* in the direction of the crack path starting at the crack tip). A crack approximately 4 mm long was introduced with a crack opening of around 10 μm near the mouth of the crack, falling more or less linearly to zero at the (sharp) crack-tip; see Fig. 2a. The crack follows the basal planes of the hexagonal Ti_2_AlC randomly oriented lamellar grains resulting in a zigzag mode, leaving local smooth cleavage fracture surfaces. Crack deflection perpendicular to the basal planes results in a stair-type fracture surface^34^. Local crack branching and crack bridging as well as grain pull-out are known features of this material. The combination of several deformation and cracking mechanisms operating in parallel is held responsible for its high toughness. The local variation in crack opening reflects the effect of the variation in grain orientations along the crack path on the local deformation and failure processes.

**Figure 3:**
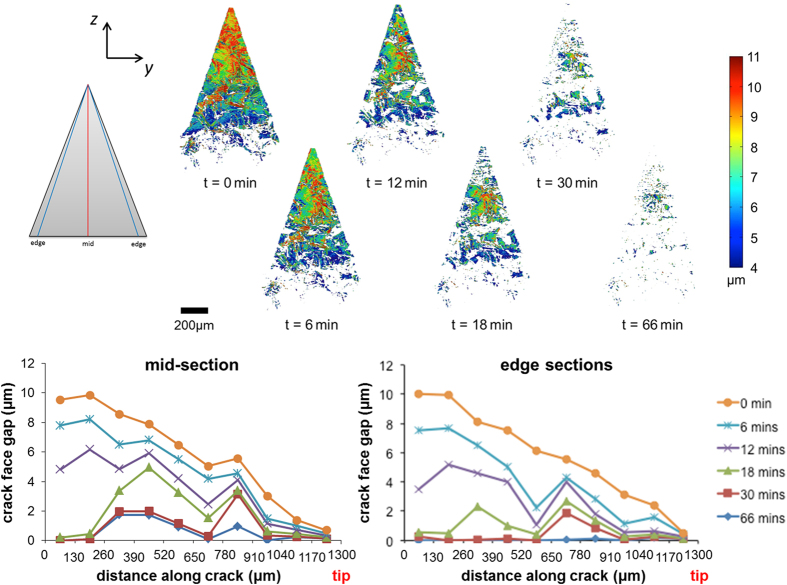
Planar views of the 3D datasets segmented to show the first crack in Ti_2_AlC both initially and at different stages of healing at 1500 K in air. This first crack (cf. Fig. 2) has a length of 1.2 mm. The superimposed colour map represents the crack face gap (CFG). The direction of crack growth is from top (mouth) to bottom (crack-tip) in the images. The graphs show the remaining opening of the crack having a length of 1.2 mm as a function of healing time for virtual sections in the middle and towards the edges of the crack (averaged over both edge trajectories).

After the crack was formed, the sample was heated to a temperature of approximately 1500 K in air and repeated tomography scans were recorded over 66 minutes of isothermal exposure (see Supplementary information Video S2: A sequence of successive planar tomographic sections of the *healed crack* in the direction of the crack path starting at the crack tip). Figure 3 shows graphs of the variation in crack face gap (CFG) across the crack faces in the initial state and after 6, 12, 18, 30 and 66 minutes, respectively. Here we use the term crack face gap to quantify the local distance between the crack faces. It is measured in the same way as crack opening displacement (COD) which is a term regularly used in fracture mechanics. Since the gap between the faces during repair is not simply related to the toughness of the material, we have used the term CFG to indicate a parameter varying locally along the length of the crack. When the crack face gap reaches a value of zero, the crack has been fully filled by a reaction product. By segmenting the crack in 3D for the duration of the healing time, a more complete analysis of the progression, and importantly the areal coverage, of crack filling can be conducted. Due to the high spatial resolution of the image acquisition and therefore of the CFG measurements, the local CFG can be extracted along a chosen trajectory from the mouth to the tip of the crack as a function of healing time. In Fig. 3 the variation in CFG along the crack centreline and along lines near both edges are considered (averaging over 5 voxels laterally at each point along each line). The edge trajectories give information on crack filling behaviour where oxygen can easily access the crack. The central trajectory gives information on shielding effects due to early local crack filling. These two graphs in Fig. 3 clearly show that the crack is significantly healed, not only near the crack edges where the oxygen enters the crack, but also along the centreline. Most importantly, a comparison of the two graphs shows that the gap between the crack faces closes more or less uniformly across its width, although the closing along the centreline was marginally slower leaving a small internal region of unhealed crack. After 30 minutes of healing a connected network of fully filled regions was found everywhere along the crack, and both crack mouth and crack tip regions were fully healed after a further 36 minutes of exposure. Small islands where the crack had not fully healed were found only in the centre of the crack. Clearly these were sealed off from the oxidizing environment during healing.

After this healing treatment, the sample was slowly cooled to 1000 K, reloaded and re-cracked. Remarkably, the second crack did not form at the location of the first healed crack but on the opposite site of the chevron; see Fig. 2b. This suggests that the adhesion between the oxides in the crack gap and the Ti_2_AlC matrix is strong and that it is energetically more favourable to initiate a new crack in pristine material than it is to re-open the healed crack.

**Figure 4:**
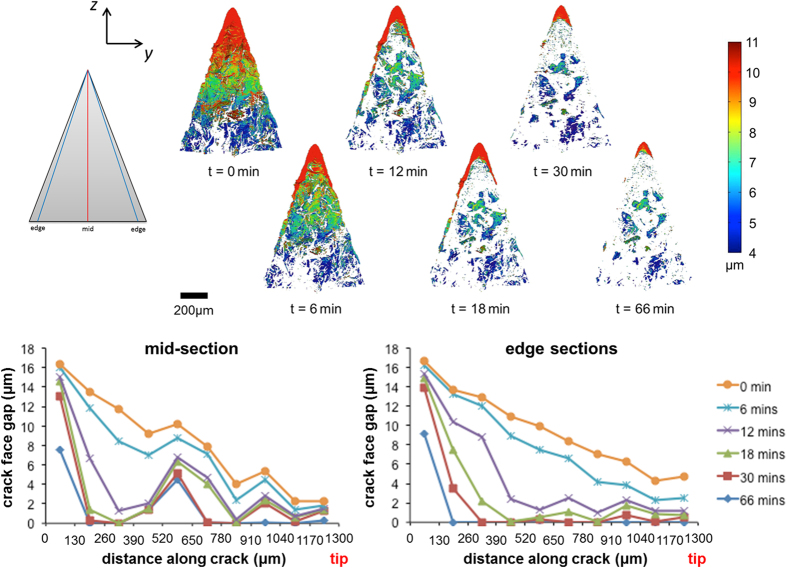
Planar views of the 3D datasets segmented to show the second crack in Ti_2_AlC both initially and at different stages of healing at 1500 K in air. This second crack (cf. Fig. 2) has a length of 1.3 mm. The superimposed colour map represents the crack face gap (CFG). The direction of crack growth is from top (mouth) to bottom (crack-tip) in the images. The graphs show the remaining opening of the crack having a length of 1.3 mm as a function of healing time for virtual sections in the middle and towards the edges of the crack (averaged over both edge trajectories).

The second crack had a crack length comparable to the first, but there was a larger initial crack opening near the mouth of the crack (viz. 17 versus 10 μm); see Fig. 4. The healing process of this second crack upon exposure to 1500 K proceeded in a similar manner to the first crack since both cracks were formed in pristine material; thus, there was a more or less uniform reduction of the crack face gap and early closing of regions where the gap was initially the smallest.

**Figure 5:**
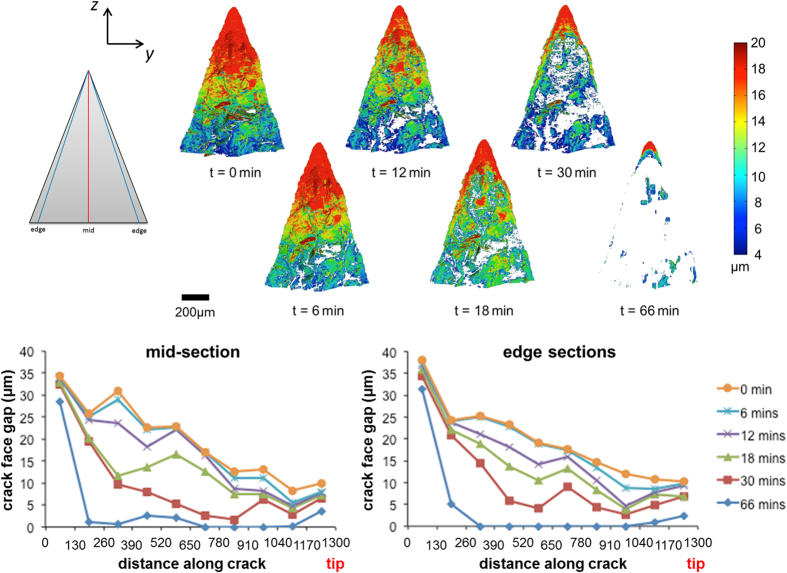
Planar views of the 3D datasets segmented to show the third, re-cracked healed second, crack in Ti_2_AlC both initially and at different stages of healing at 1500 K in air. This third, re-cracked healed second crack (cf. Fig. 2), has a length of 1.3 mm. The superimposed colour map represents the crack face gap (CFG). The direction of crack growth is from top (mouth) to bottom (crack-tip) in the images. The graphs show the remaining opening of the crack having a length of 1.3 mm as a function of healing time for virtual sections in the middle and towards the edges of the crack (averaged over both edge trajectories).

After the second crack healed, the sample was cooled again and re-cracked at a temperature of 1000 K. Given the fact that the first crack effectively received a double healing treatment and that the mouth of the second crack had not been filled completely (Fig. 4), it is not surprising that the third crack followed the path of the second; see Fig. 2d. The crack opening for the re-cracked (third) crack (Fig. 5) is larger than for the original (second) crack (Fig. 4) made in the pristine material (viz. 35 versus 17 μm). It is interesting and very encouraging to note that upon re-cracking substantial further crack filling was observed upon re-exposure to 1500 K even leading to small regions with complete crack filling in the crack interior; see the time dependent sequences of Fig. 5. In the supplementary information Videos are included that show the healing of these three cracks as time-lapse segmented 3D datasets; see Videos S3, S4 and S5.

### Crack healing kinetics

The *local* crack healing kinetics are determined from the measured evolution of the CFG, cf. Figs 3, 4 and 5. At each location within the crack gap, the amount of healing can be expressed as:


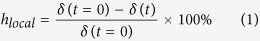


where *δ(t)* is the CFG at time *t*. The change in CFG follows from the oxidation kinetics, hence:


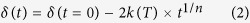


where *k(T)* is the temperature *T* dependent rate constant and *n* is the rate exponent, respectively. For alumina forming MAX phases *n* equals about 3, because in general, the oxidation obeys a cubic growth rate law^35^. However, for the Ti_2_AlC MAX phase studied here a value of about 4 was observed^15^. The growth of oxide in the gap of the crack proceeds as long as the oxide surface is accessible to the external environment. Thus, when the crack gap is sealed off, or when the oxide at either side closes the crack gap, the oxide growth ceases.

The *global* crack healing kinetics is determined from change in the volume *V(t)* of the crack as it fills with oxide. This volume change is determined from the 3D datasets by counting the number of voxels that represent the crack gap at a given time, t, hence:


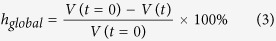


Since the change of the volume *V(t)* of the crack is related to the change of the CFG according to Eq. (2), the global healing kinetics may show a similar trend to the oxidation kinetics.

**Figure 6:**
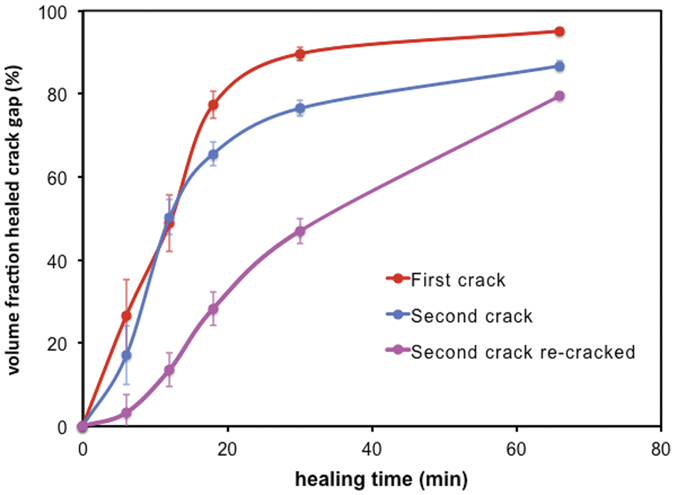
Crack healing kinetics in Ti_2_AlC at 1500 K in air, presented in terms of volume fraction of the initial crack gap filled with oxide as a function of time. The first and second crack reflect healing in pristine material, while the third crack shows healing of the re-cracked and re-healed second crack (cf. Fig. 2).

As shown in Fig. 6, both cracks in the pristine material (i.e. the first and second cracks, cf. Fig. 2) display essentially identical crack filling kinetics, showing the reproducibility of the experiment and data reconstructions. In agreement with the macro measurements on the oxidation kinetics of Ti_2_AlC^15^, the initial rate of oxide growth for crack gap filling is fast, forming a closed alumina layer on the fracture surfaces. The healing process is subsequently retarded by the presence of the oxide layer covering the fracture surfaces. This is most significant in the case of the third crack (at the location of the healed second crack, cf. Fig. 2). The rate of local oxide growth is reduced because of the existing oxide formed from the first healing cycle. For the third crack the amount of crack filling after 1 hour at 1500 K was about 80%, while the first and second cracks in the pristine material were filled to about 90% at this stage.

## Discussion

The results presented here demonstrate the usefulness of *in situ* 4D (time-lapse) tomographic imaging when studying crack repair in a self-healing MAX phase materials. The change in crack face gap is observed with a high spatial resolution and has yielded the local, as well as the average, evolution of crack healing in pristine material and for the healing of a crack re-formed along a previously healed crack. For the first time we are able to resolve the spatial and temporal local crack filling kinetics.

Previous work on multiple crack healings show that the strength fully recovers when a crack damaged Ti_2_AlC MAX phase material is exposed to air at high temperatures^4^. However, the toughness of the composite Ti_2_AlC matrix with oxide filled healed cracks reduces upon subsequent healing cycles. As observed for the healing of a re-cracked healed crack (Fig. 6), it becomes more difficult to heal a crack that is previously filled with oxide, and the ‘scar’ created becomes wider; see Fig. 5. Although the Al of Ti_2_AlC is consumed during crack healing, the diffusion of Al in Ti_2_AlC is fast^15^ and deviation from its stoichiometric composition can be large (up to 50%)^16^. Hence in multiple healing events the base Ti_2_AlC material effectively acts as an infinite reservoir of the Al healing agent. Finally, it should be mentioned that Ti_2_AlC is stable up to 1650 K^36^ and no grain growth or compositional changes occur upon healing at 1500 K. Post mortem analysis of the sample confirmed that besides surface oxidation in the crack nor the microstructure nor the composition was changed.

The information presented here is crucial for constructing models predicting local damage and healing under practical operating conditions and also in the interpretation of healing kinetics as a function of initial damage topology and material compositions. This study opens new avenues for development and design of self-healing high temperature ceramics, not only for MAX phase materials when optimizing their composition and microstructure for crack healing, but also for self-healing oxide ceramics with sacrificial particles where their composition, size and distribution are crucial^37^.

## Methods

### Material preparation

A Ti_2_AlC disc was synthesized by hot pressing a dry mixture of Ti, Al and C powders (with a particle size of about 50 μm and purity better than 99%) at 1625 K under 30 MPa of pressure for 4 hours in Ar. For the X-ray tomographic microscopy experiments, the samples were machined by electro discharge machining (EDM) to cylinders with a diameter of 2.6 mm and 7 mm length into which a chevron-notch geometry was cut; see Fig. 1b. The tip of the chevron has a thickness of 0.4 mm, a height of 3.65 mm, and a top angle of 39.2°; it is located 1.65 mm below the upper surface of the cylinder.

### Mechanical testing rig and laser-based heating system

Figure 1 shows the setup of the mechanical testing rig^32^ with the laser-based heating system^33^ incorporated into its frame. The sample sat on the lower platen inside an alumina sleeve of internal diameter 3 mm and wall thickness of 1 mm. The outer surface of the alumina sleeve was covered with blackbody paint in order to more efficiently absorb the power of the lasers and subsequently transfer heat to the sample, thereby acting as a small furnace. It also served as a sample holder and air between the sleeve and the sample acted to dissipate the heat and provide the oxidizing environment. The temperature of the sample was controlled using a single-wavelength pyrometer (Optris GmbH, Germany), which measures the temperature of the material in its line-of-sight (i.e., the alumina sleeve covered with black paint). The temperature during the healing experiment was constant to within ± 10 K.

A crack was grown in the sample in a controlled manner by lowering a wedge, fixed to the upper platen, slowly into the notch with a displacement rate of 5 μm/min. The wedge tip had an angle of 10° and the wedge itself was machined from a Ni superalloy (CMSX-4) such that it could withstand the high working temperatures of the experiment. Once the wedge was in contact with the sides of the notch, observed by monitoring a small force reading (typically 2 N), the wedge was lowered continuously whilst monitoring both the force and a live preview of the X-ray projection of the sample; together, these tools provided the means to identify crack initiation. Crack growth was then performed at a temperature of 1000 K in order to avoid further crack opening during the initiation of heating, during which the wedge remained in the chevron notch. Once a crack had initiated, corresponding to a force of 60 N, the wedge was driven further into the sample until the crack grew to a length of around the height of the field-of-view of the detector (approximately 1.5 mm). With the wedge remaining in position, the laser power was increased to a corresponding temperature of 1500 K at a rate of 50 K/min. Radial temperature uniformity was achieved through continuous rotation during heating after the crack formation. The sample was then isothermally held at 1500 K for about 80 minutes whilst continuous tomography scans were recorded at user-defined intervals. Two further crack growth and healing cycles were subsequently performed.

### X-ray tomographic microscopy

Measurements were performed at the TOMCAT beamline^32^,^33^ of the Swiss Light Source at the Paul Scherrer Institut (Villigen, Switzerland). Projection images were collected using a pco.EDGE sCMOS camera coupled to a long-working distance microscope (Optique Peter, France) with 10x magnification. Using a 50% power filter on the polychromatic x-rays, the exposure time for a single projection was 15 ms. A full three-dimensional dataset comprised of 1001 projections over 180° was acquired in 15 s followed by a user-defined 30 s interval, resulting in one scan every 45 s. The isotropic pixel size was 0.65 μm giving a field-of-view 1.7 mm wide by 1.4 mm high. The central region of the sample, centered on the chevron notch, was imaged. Tomographic reconstructions were achieved using a re-gridding Fourier transform-based reconstruction algorithm^38^. Image processing and visualisation were performed using Avizo (Visualisation Sciences Group).

In order to quantify the crack face gap (CFG) a median filter was applied to the original X-ray tomographic reconstructions to reduce the background noise. The cracked region in each tomographic slice was then segmented by thresholding. The gap across the faces (CFG) was measured as a function of position by counting the number of pixels occupied by the crack at each location perpendicular to the crack growth direction. In this way the current gap between the crack faces can be mapped for each crack at any stage in the repair process.

## Additional Information

**How to cite this article**: Sloof, W. G. *et al.* Repeated crack healing in MAX-phase ceramics revealed by 4D *in situ* synchrotron X-ray tomographic microscopy. *Sci. Rep.* **6**, 23040; doi: 10.1038/srep23040 (2016).

## Supplementary Material

Supplementary Video S1

Supplementary Video S2

Supplementary Video S3

Supplementary Video S4

Supplementary Video S5

Supplementary Information
