# Supplementary material for: Repeated crack healing in MAX-phase ceramics revealed by 4D in situ synchrotron X-ray tomographic microscopy
Source: Sci Rep. 2016 Mar 14;6:23040. doi: 10.1038/srep23040 (PMC4789783; doi:10.1038/srep23040)
Supplement: Supplementary Information [file srep23040-s6.pdf]

Repeated crack healing in MAX-phase ceramics revealed by 4D in situ synchrotron X-ray tomographic microscopy

Willem G. Sloof[1], Samuel A. McDonald[2], Ruizhi Pei[2], Julie L. Fife[3], Lu Shen[1], Linda Boatemaa[1], Ann-Sophie Farle[1], Kun Yan[2], Xun Zhang[2], Sybrand van der Zwaag[4], Peter D. Lee[2,5], and Philip J. Withers[2,5]

[1]Department of Materials Science and Engineering, Delft University of Technology, Mekelweg 2, 2622 CD, Delft, The Netherlands. [2]School of Materials, Manchester University M13 9PL, UK. [3]Swiss Light Source, Paul Scherrer Institut, 5232 Villigen PSI, Switzerland. [4]Faculty of Aerospace Engineering, Delft University of Technology, Kluyverweg 1, 2629 HS, Delft, The Netherlands. [5]Research Complex at Harwell, Didcot, Oxfordshire, OX11 0FA, UK.
Correspondence and requests for materials should be addressed to W.G.S. (email: W.G.Sloof@TUDelft.nl).

Supplementary information

Video S1: A sequence of successive planar tomographic sections of the *initial crack* in the direction of the crack path starting at the crack tip.

Video S2: A sequence of successive planar tomographic sections of the *healed crack* in the direction of the crack path starting at the crack tip.

Video S3: Healing of the first crack; time-lapse segmented 3D datasets.

Video S4: Healing of the second crack; time-lapse segmented 3D datasets.

Video S5: Healing of the reopened and previously healed second crack; time-lapse segmented 3D datasets.
